# Case Report: Multimodality imaging features and serial metabolic changes on ^18^ F-FDG PET/CT in a dog with granulomatous hepatitis

**DOI:** 10.3389/fvets.2026.1769462

**Published:** 2026-06-30

**Authors:** Sunghwa Hong, Seungjo Park, Sang-Kwon Lee, Kyoung-oh Cho, Jihye Choi

**Affiliations:** 1 Department of Veterinary Medical Imaging, College of Veterinary Medicine, Seoul National University, Seoul, Republic of Korea; 2 Department of Veterinary Medical Imaging, College of Veterinary Medicine and BK21 FOUR Program, Chonnam National University, Gwangju, Republic of Korea; 3 Department of Veterinary Medical Imaging, College of Veterinary Medicine, Kyungpook National University, Daegu, Republic of Korea; 4 Laboratory of Veterinary Pathology, College of Veterinary Medicine, Chonnam National University, Gwangju, Republic of Korea

**Keywords:** canine, computed tomography, GH, positron emission tomography, target sign

## Abstract

Granulomatous hepatitis is an uncommon inflammatory liver disease that can mimic hepatic malignancy on diagnostic imaging. A 12-year-old spayed female Cocker Spaniel presented with multiple target-like hepatic and splenic lesions showing peripheral enhancement on triple-phase CT and markedly increased ^18^F-FDG uptake on PET/CT (SUVmax 11.2), raising strong suspicion for malignancy. However, histopathology confirmed granulomatous hepatitis. Serial PET-CT performed 4 months later demonstrated decreased metabolic activity, supporting a chronic inflammatory process. This case highlights the potential role of serial PET-CT beyond malignancy assessment, demonstrating its utility for evaluating metabolic activity and monitoring therapeutic response over time in inflammatory hepatic disease such as active granulomatous hepatitis.

## 1 Introduction

Granulomatous hepatitis is an uncommon but clinically important inflammatory liver disease characterized by histiocytic infiltration, variable numbers of neutrophils and plasma cells, and disruption of normal lobular architecture (1, 2). Granulomatous hepatitis may present with hyperechoic periportal bands, hyperechoic nodules with hypoechoic rims, multifocal hepatic nodules, and heterogeneous parenchymal changes (3).

Diagnostic imaging plays an important role in the evaluation of canine hepatic disease; however, the reported imaging features of granulomatous hepatitis are limited in veterinary medicine (3). Ultrasonographic findings may include altered parenchymal echogenicity, and focal or multifocal nodular lesions, which can overlap substantially with those of hepatic neoplasia. Computed tomography (CT) mainly focused on nonspecific morphologic changes rather than contrast-enhancement characteristics.

Positron emission tomography–computed tomography (PET-CT) using ^18^F-fluorodeoxyglucose (FDG) is utilized for tumor detection, staging, treatment monitoring, and particularly for detecting metastatic lesions (4–8). FDG is a glucose analog radiotracer that enables assessment of tissue glucose metabolism. Because many malignant tumors exhibit rapid growth and increased metabolic demand, they typically demonstrate higher glucose utilization than normal tissues, resulting in increased FDG accumulation (9–11). Moreover, the standardized uptake value (SUV) provides a semiquantitative measure of FDG uptake and higher SUVmax values are associated with aggressive biological behavior, increased cellular proliferation, and poorer prognosis in various malignant tumors. Consequently, markedly elevated SUVmax values generally raise clinical concern for high-grade malignancy. However, FDG uptake is not specific to neoplasia and may also be increased in inflammatory conditions due to heightened metabolic activity of activated inflammatory cells (12–14). In hepatic pathology, PET-CT can provide functional information complementary to morphologic imaging, particularly for detecting metastatic disease and evaluating hepatic involvement in systemic or inflammatory disorders (15). However, increased hepatic FDG uptake should be interpreted cautiously because it may occur in both neoplastic and non-neoplastic conditions.

Granulomatous hepatitis in dogs has been described primarily from a histopathologic perspective, however diagnostic imaging features area limited only in ultrasound and CT examinations (1, 2). Multiphasic CT and FDG PET-CT, as well as the serial examination of imaging findings have not been reported yet. Therefore, this study described the multimodal imaging features of granulomatous hepatitis in a dog, including ultrasonography, triple-phase CT, and serial PET-CT.

## 2 Case description

A 12-year-old spayed female Cocker Spaniel weighing 12 kg was referred to Chonnam National University Veterinary Teaching Hospital with a 1-week history of anorexia, lethargy and a clinical suspicion of splenic tumor. The body temperature 39.3 °C, heart rate 102 beats/min, and respiratory rate 42 breaths/min. Complete blood count showed leukocytosis (29.73 × 10^9^/L; reference 5.05–16.76 × 10^9^/L), neutrophilia (23.89 × 10^9^/L; reference 2.95–11.64 × 10^9^/L), and monocytosis (2.26 × 10^9^/L; reference 0.16–1.12 × 10^9^/L). Serum biochemistry revealed elevated alanine aminotransferase (ALT, 133 U/L; reference 10–100 U/L), alkaline phosphatase (ALP, 580 U/L; reference 23–212 U/L), and markedly increased C-reactive protein (CRP, 183 mg/L; reference <1 mg/L).

Abdominal radiographs showed hepatomegaly, splenomegaly, and mineralization of both kidneys. No abnormalities were detected on thoracic radiographs.

On abdominal ultrasonography (Alpha 7, Hitachi Aloka Medical, Tokyo, Japan), multiple hepatic masses were observed. In the quadrate and left lateral lobes, the masses exhibited a target-like appearance, characterized by hyperechoic centers surrounded by hypoechoic rims, and measured 5–30 mm in diameter (Figures 1A)–(Figures C). Additional smaller hypoechoic nodules (2.3–10 mm) were observed in the right lobe. The intervening hepatic parenchyma was diffusely hyperechoic with fine echotexture, and mild dilation of intrahepatic vessel was found. The left hepatic margin appeared mildly rounded. On color Doppler imaging, no distinct blood flow signals were identified apart from the intralesional vessels (Figure 1D). The hepatic lymph node was enlarged and hypoechoic (Figure 1E). Multiple hypoechoic splenic nodules measuring ≤ 5 mm in diameter were identified, and one nodule measuring 8.3 × 7.2 mm exhibited a hyperechoic center with a hypoechoic peripheral rim and show no detectable blood flow signal on color Doppler imaging (Figure 1F), (Figure 1G). Based on these findings, diffuse infiltrative tumor such as lymphoma or metastatic tumor was considered, with potential involvement to the hepatic lymph nodes.

**Figure 1:**
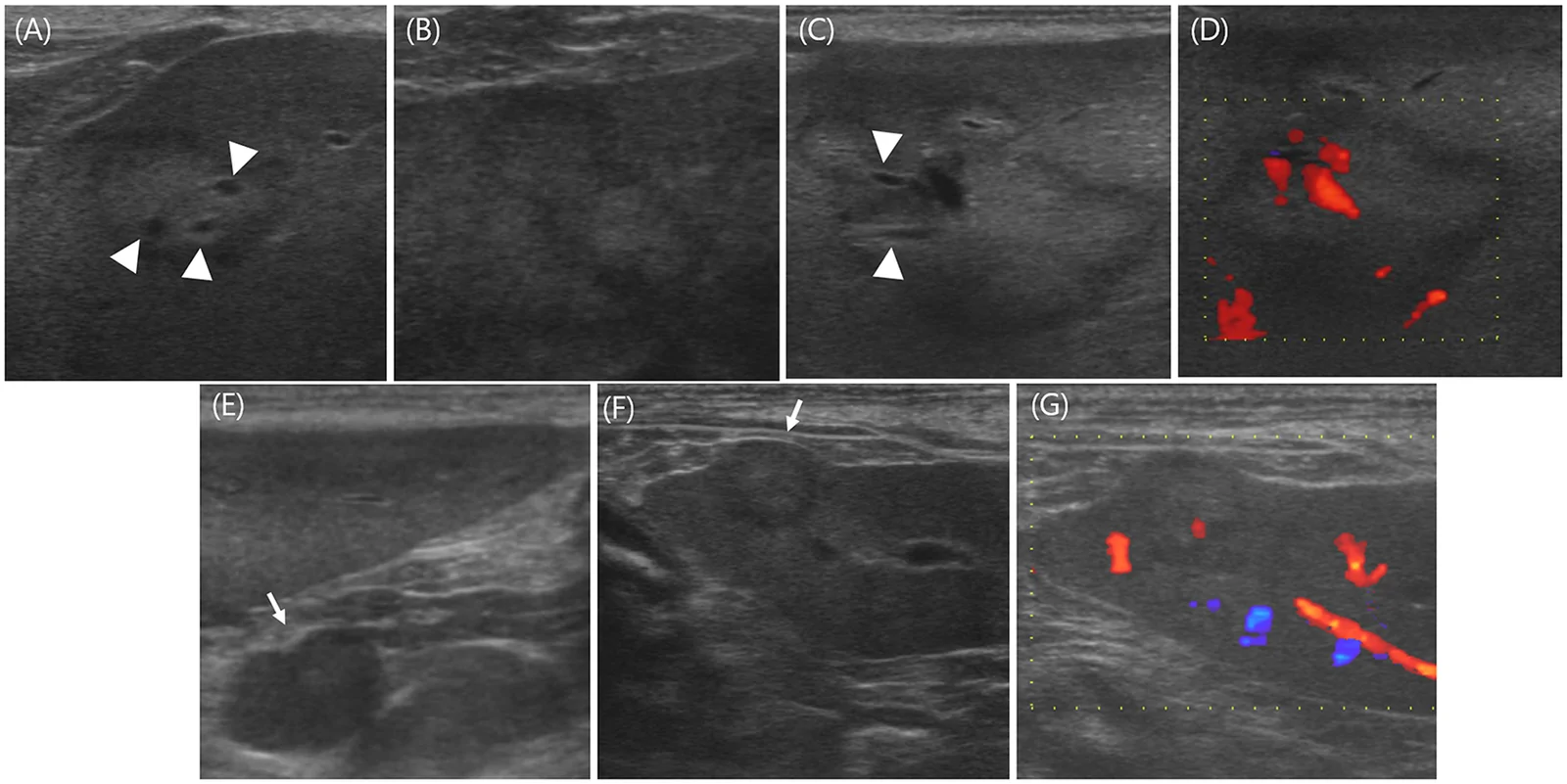
Ultrasonographic images of the quadrate lobe and left lateral hepatic lobe **(A–C)** and corresponding color Doppler image **(D)**, the hepatic lymph node **(D)** and spleen **(F, G)**. Multiple hepatic masses, ranging from approximately 5 to 30 mm in diameter, are present and exhibit a hypoechoic peripheral rim surrounding a hyperechoic central region **(A–C)**. The hyperechoic centers commonly contain intralesional vascular structures (arrowhead). The adjacent hepatic parenchyma demonstrates diffusely increased echogenicity with a fine echotexture. On color Doppler imaging, no detectable blood flow signals are observed within the masses apart from the intralesional vessels **(D)**. **(E)** The hepatic lymph node is enlarged and appears distinctly hypoechoic (arrow). **(F, G)** A splenic nodule (arrow), measuring 8.3 × 7.2 mm, is identified with a hyperechoic center and a hypoechoic peripheral rim. No detectable blood flow signals are observed**(G)** within the nodule on color Doppler imaging.

Computed tomography (CT) was performed using a 16-slice scanner (Somatom Emotion 16, Siemens, Forchheim, Germany) with the following acquisition parameters: 130 kVp, 120 mAs, a pitch of 1 mm, and 2 mm slice thickness. The patient was positioned in ventral recumbency and scanned in a head-first orientation. Triple phasic contrast CT images were acquired following intravenous administration of iohexol (Omnipaque^®^, GE Healthcare, China) at a dose of 880 mg I/kg and an injection rate of 3 mL/s. The arterial and portal venous phases were obtained using a test-bolus technique, with the region of interest placed in the abdominal aorta and hepatic vein to determine the timing of peak enhancement. The exact scan times after contrast administration for the arterial and portal venous phases were not recorded. Equilibrium phase images were acquired approximately 3 min 30–40 s after contrast administration. On pre-contrast images, multiple hypoattenuating, mass-like lesions were identified throughput the liver. These lesions were irregular in shape and had indistinct margins. On post-contrast images, the lesions were consistently distributed along the distal branches of both the hepatic arteries and portal veins and formed multilobulated, mass-like structures. On the arterial phase, the lesions displayed linear peripheral enhancement with minimal to no enhancement in the central portions (Figure 2A), (Figure E). Then, most lesions became more conspicuous and showed a clearer target-like appearance, characterized by distinctly hypoattenuating rims relative to the surrounding parenchyma on the portal venous phase (Figures 2B),(Figure F), (Figure I). Several additional smaller nodules showing similar target-like enhancement patterns were present. An 18 × 13 × 12 mm, homogeneous hyperattenuating mass was also observed in the quadrate lobe (Figure 2I). During the equilibrium phase, the central portion of the target-like hepatic lesions became slightly hyperattenuating compared with the adjacent liver parenchyma (Figure 2C), (Figure G). In contrast, the hyperattenuating mass in the quadrate lobe was no longer conspicuous on equilibrium phase, supporting the interpretation of a transient hepatic attenuation difference (THAD).

**Figure 2:**
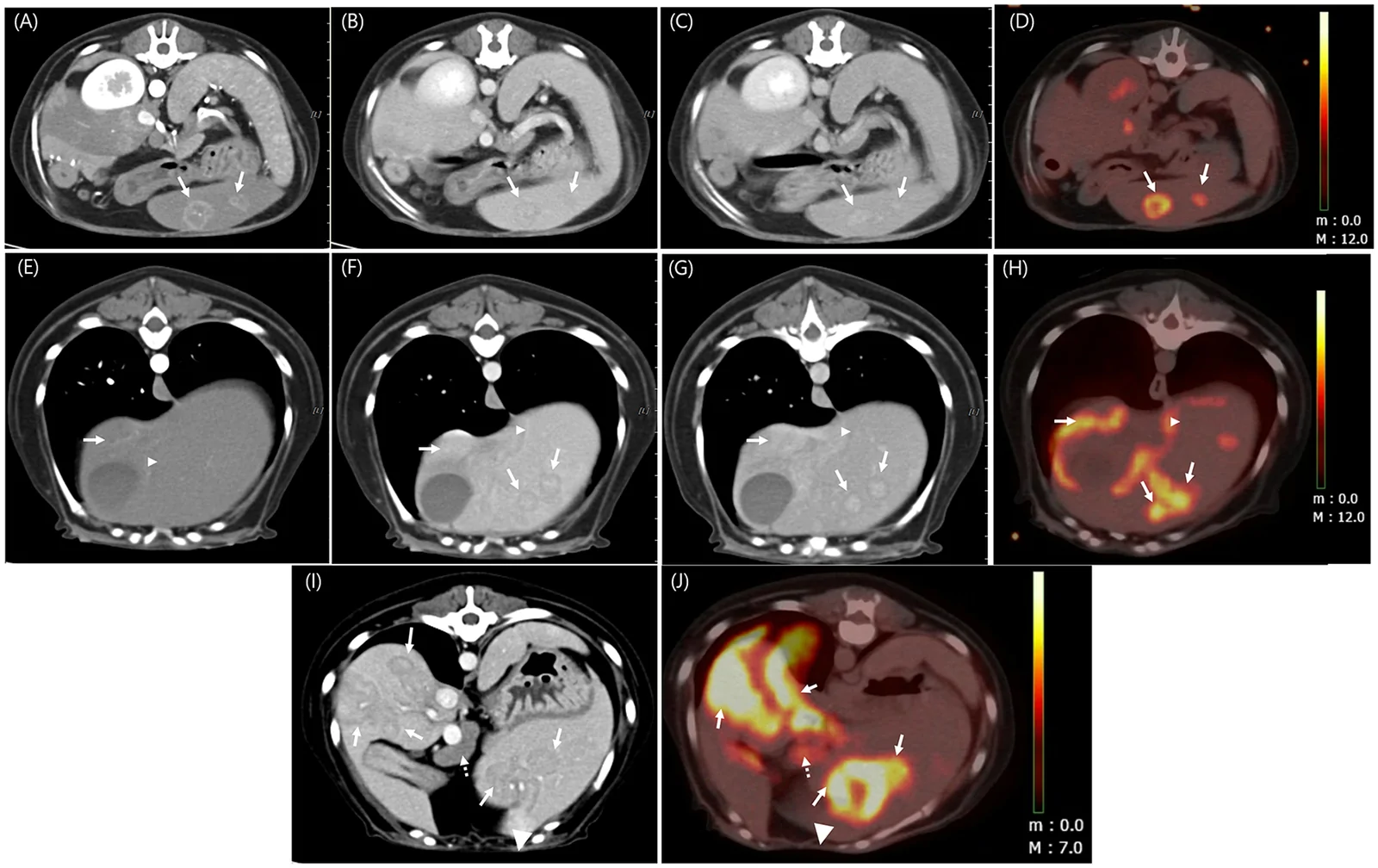
Transverse images of hepatic lesions and lymph node on the arterial **(A, E)**, portal venous **(B, F, I)**, and equilibrium **(C, G)** phases of computed tomography and on positron emission tomography–computed tomography (PET-CT) following administration of ^18^F-fluorodeoxyglucose **(D, H, J)**. **(A, E)** In the arterial phase, multiple hepatic masses were observed throughout the parenchyma, each demonstrating marked peripheral enhancement with an internal hepatic arterial branch visible at the center (arrows). **(B, F)** In the portal venous phase, the lesion margins appeared hypoattenuating, while the centers become isoattenuating relative to the surrounding liver parenchyma (arrows). **(C, G)** In the equilibrium phase, the lesion centers appeared hyperattenuating compared with the surrounding liver, whereas the rims remain hypoattenuating, similar to the portal venous phase (arrows). **(D, H)** PET-CT images at corresponding levels demonstrate hypermetabolism within the hepatic masses, with maximum standardized uptake value (SUVmax) measurements ranging from 2 to 11.2 (arrows). The standardized uptake value scale was displayed from 0 to 12. **(I)** Multiple target-like hepatic lesions are visible throughout the liver in the portal venous phase (arrows), and a distinctly hyperattenuating mass is observed along the ventral margin of the left lateral hepatic lobe (arrowhead). The hepatic lymph node is markedly enlarged (dashed arrow). **(J)** On PET-CT at the corresponding level, the multiple target-like hepatic lesions exhibit high SUVmax, whereas the hyperattenuating mass (arrowhead) shows an SUVmax similar to that of the surrounding hepatic parenchyma, rendering it inconspicuous and supporting the interpretation of a transient hepatic attenuation difference. The hepatic lymph node (dashed arrow) demonstrates mildly increased metabolic activity with an SUVmax of 2.9.

In the spleen, multiple hyperattenuating nodules and a single hypoattenuating nodule observed during the arterial and portal venous phases. The multiple hyperattenuating nodules was no longer visible in the equilibrium phase, consistent with transient enhancement commonly associated with extramedullary hematopoiesis. In contrast, the hypoattenuating nodule at the splenic tail remained unchanged across all phases.

In addition, two distinctly enlarged hepatic lymph nodes, measuring 10 × 12 × 24 mm were observed (Figure 2I), and mild enlargement of the gastric and pancreaticoduodenal lymph nodes was also observed.

Ultrasound-guided fine-needle aspiration of the hepatic nodules revealed vacuolar changes, mononuclear cell infiltration, anisocytosis, anisokaryosis, and inflammatory cells. Differential diagnoses included steroid-associated hepatopathy, nodular hyperplasia/regeneration, or inflammatory hepatitis.

However, the imaging findings, particularly the presence of multiple target-like hepatic masses throughout the parenchyma and the distinctly peripheral enhancement pattern on arterial-phase CT images, were not consistent with the typical enhancement appearance of nodular hyperplasia. In addition, the markedly enlarged, rounded hepatic lymph nodes raised concern for malignancy.

Therefore, positron emission tomography–computed tomography (PET-CT) was conducted to further assess tumor metabolism and malignancy potential, identify a potential primary neoplasm, and exclude the possibility of metastatic disease from an occult primary tumor. The dog was fasted for 12 h and blood glucose was measured immediately before imaging. Anesthesia was induced and maintained with zolazepam/tiletamine (1.5 mg/kg, IM) and medetomidine (0.03 mg/kg, IM). After induction of anesthesia, Radiotracer, ^18^F-FDG (1.83 mCi; 11 MBq/kg) was administered intravenously. The dog was maintained under anesthesia throughout the 50-min uptake period. Then, PET-CT was performed using a scanner (Discovery 600, GE Healthcare, USA). A non–contrast-enhanced CT scan was acquired using an 8-slice helical scan protocol (120 kVp; 80 mAs; 3.79-mm slice thickness), and CT data were used for attenuation correction of the PET images and anatomical localization. The emission scan portion of the PET study was subsequently performed, and the images were reconstructed using a 3.3 mm slice thickness with the standardized uptake value (SUV) scale set to 0–7 or 0–12 to optimize visualization of hypermetabolic lesions (Figure 2D), (Figure 2H). The SUV scale was adjusted in accordance with the metabolic characteristics of the target lesions to ensure optimal conspicuity. The mean standardized uptake value (SUVmean) of the hepatic background was 2.1. The target-like hepatic masses showed marked hypermetabolism, with maximum standardized uptake value (SUVmax) ranging from 2 to over 11.2 (Figure 2D), (Figure 2H), (Figure 2J). The hepatic lymph nodes showed mild hypermetabolism, with an SUVmax of 2.9, raising suspicion for malignancy of the hepatic lesions. The hepatic lesion previously suspected to represent a THAD demonstrated SUVmax value comparable to that of the surrounding normal liver parenchyma (Figure 2J). All splenic nodules, including the hypoattenuating lesion, showed SUV values within a benign range. No additional abnormalities suggestive of a primary malignant neoplasm were identified on whole-body evaluation.

An ultrasound-guided percutaneous Tru-cut liver biopsy were performed the day after the PET-CT examination, and samples from multiple hepatic lesions were obtained for definitive diagnosis. Histopathologic evaluation of samples revealed chronic multifocal to confluent granulomatous and lesser neutrophilic plasmacytic interstitial hepatitis, accompanied by severe chronic cholestasis, replacing approximately 40%−50% of the hepatic parenchyma (Figure 3). The inflammatory infiltrates consisted predominantly of mature histiocytes, with fewer neutrophils, plasma cells, and occasional eosinophils, forming confluent nodules that effaced the normal lobular architecture. No neoplastic cells, mitotic figures, or infectious organisms were identified.

**Figure 3:**
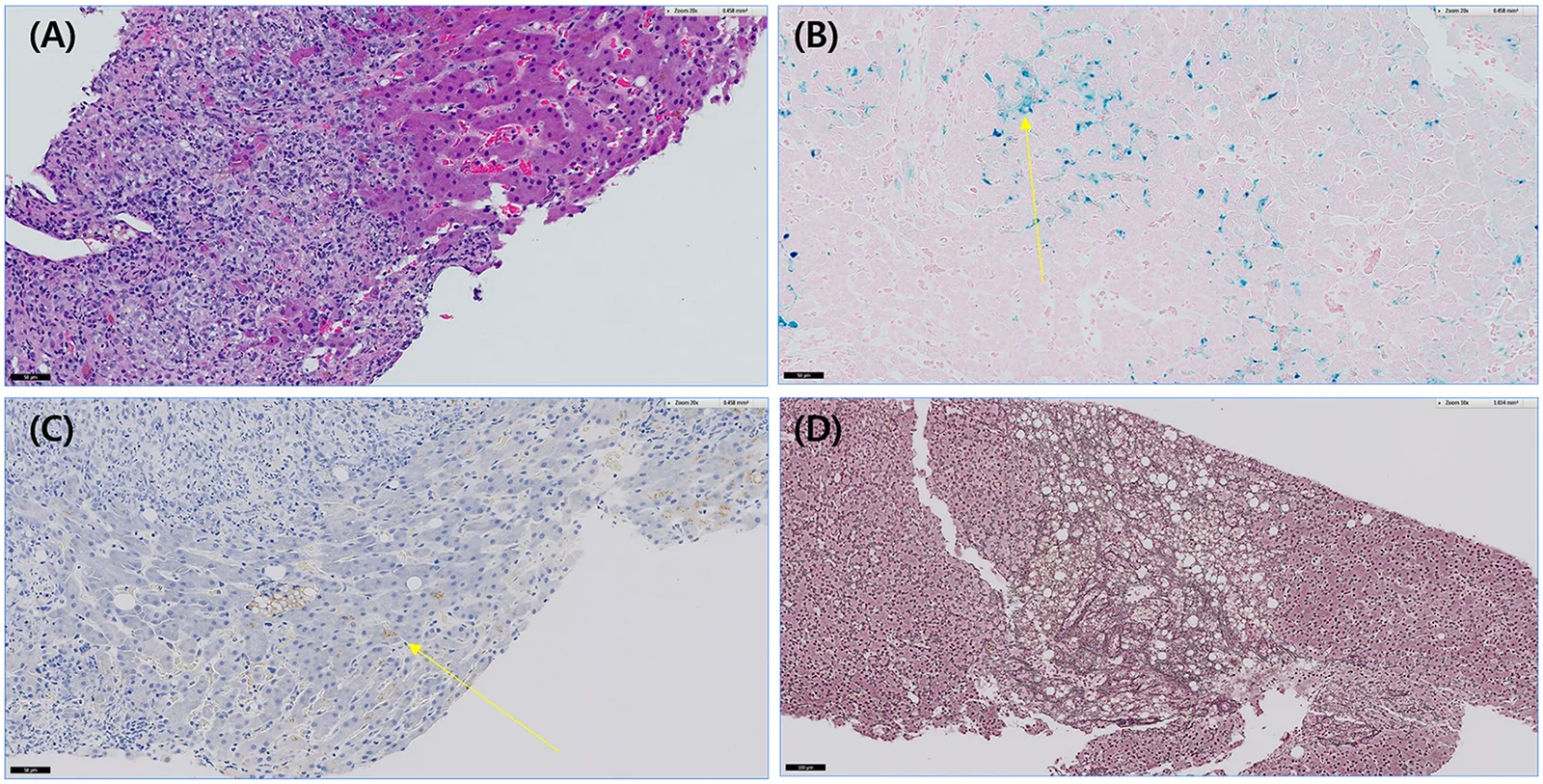
Histopathologic examination revealed chronic multifocal to confluent granulomatous and lesser neutrophilic plasmacytic interstitial hepatitis with severe chronic cholestasis. **(A)** Dense granulomatous and mixed inflammatory infiltrates accompanied by biliary hyperplasia and hepatocellular atrophy were observed, with extensive replacement of normal hepatic architecture by mature inflammatory cells (hematoxylin and eosin stain; 20×). **(B)** Moderate to marked accumulation of iron pigment (yello arrow) was present, primarily within inflamed and necrotic regions, whereas viable non-necrotic hepatocytes lacked iron (iron stain; 20×). **(C)** Moderate copper retention (yellow arrow) was identified, although its lobular distribution was difficult to determine due to architectural distortion from lobular atrophy, inflammation, and biliary hyperplasia (copper stain; 20×). **(D)** Increased connective tissue and fibrosis were evident throughout the hepatic lobule, predominantly in periportal areas but extending into midzonal and periacinar regions (trichrome stain; 10×).

Special stains were negative for acid-fast bacteria (Fite's stain) and fungi (GMS), effectively excluding common infectious etiologies. Additional copper staining demonstrated moderate diffuse hepatic copper accumulation involving both periportal and periacinar regions, a level exceeding that typically observed in simple cholestatic copper retention and suggestive of possible early primary copper toxicosis. Ancillary iron staining confirmed moderate to marked hemosiderin deposition within inflamed and necrotic regions, and trichrome and reticulin stains showed lobular collapse, sinusoidal compression, and increased fibrosis indicative of chronic hepatic injury.

Taken together, these findings support a diagnosis of chronic granulomatous hepatitis with marked cholestasis, copper accumulation, and significant architectural distortion, with no evidence of neoplasia.

The dog was treated with prednisolone (0.5 mg/kg), ursodeoxycholic acid (15 mg/kg), biphenyl-dimethyl-dicarboxylate (25 mg), and S-adenosylmethionine (20 mg/kg). The dog showed improvement in appetite and activity after treatment, however, serum ALT (216 U/L; reference 10–100 U/L) and ALP (771 U/L; reference 23–212 U/L) remained elevated, and increased CRP was also increased (163 mg/L; reference <1 mg/L).

A follow-up PET-CT examination was performed 4 months later to evaluate treatment response and monitor the progression or resolution of the hepatic and splenic lesions using the same acquisition protocol as the initial study. The background hepatic SUVmean was measured at 2.0. Multiple hypermetabolic lesions were persistently observed in the liver and the hepatic lymph node. The hepatic lesions showed a decrease in SUVmax from 11.2 to 8.8, and the SUVmax of the hepatic lymph node decreased from 2.9 to 1.8. Although still relatively high, this gradual reduction was more consistent with an active inflammatory process than with a progressively aggressive neoplasm. No new metastatic lesions were detected.

Ten months after the initial diagnosis, there were no remarkable changes in clinical conditions. However, serum ALT (477 U/L; reference 10–100 U/L), ALP (1866 U/L; reference 23–212 U/L), and blood urea nitrogen (BUN, 30 mg/dL; reference 7–27 mg/dL) were elevated compared with previous measurements.

At the 10-month to 1-year ultrasound follow-up, the target-like hepatic masses had changed to heterogeneously hypoechoic pattern. The liver parenchyma showed diffusely increased echogenicity with fine echotexture, and the hepatic margins had become more irregular and rounded (Figure 4). A small amount of anechoic ascites was also found. These changes were consistent with chronic hepatic cirrhosis, with the hepatic nodules representing inflammatory and fibrotic cirrhotic nodules rather than neoplastic masses, although no additional liver biopsy was performed.

**Figure 4:**
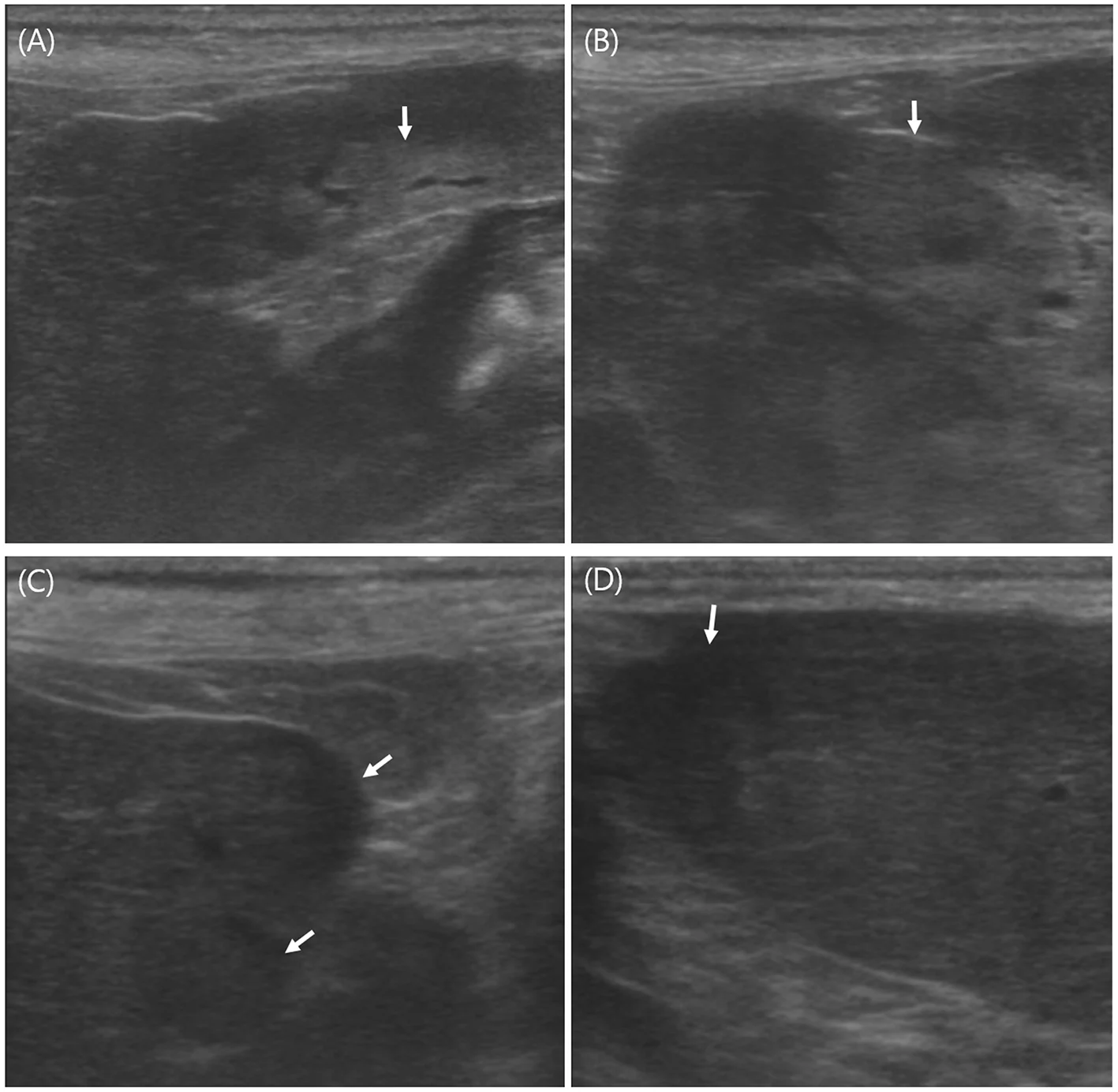
Ultrasonographic images of the caudate lobe **(A)** and left hepatic lobe **(B)** at the 10-month follow-up, and the left hepatic lobe **(C)** and right hepatic lobe **(D)** at the 1-year follow-up. **(A, B)** At the 10-month follow-up, the previously target-shaped lesions had transformed into diffuse hyperechoic nodules (arrows), and the hepatic margins appeared rounder than before. **(C, D)** At the 1-year follow-up, many of the hepatic lesions had changed into a homogeneous hypoechoic pattern (arrows), and the hepatic margins became even more rounded. Although not shown in these images, ascites was also identified.

The dog continued to receive hepatoprotective agents and prednisolone; however, clinical signs such as anorexia and lethargy waxed and waned throughout the course of management. The dog survived for 2 years and 3 months after the initial diagnosis, after which follow-up monitoring was discontinued.

## 3 Discussion

Granulomatous hepatitis can show highly variable imaging features, occasionally mimicking hepatic neoplasia on conventional and advanced imaging modalities. In the present dog, multiple target-like lesions involving both the liver and spleen, accompanied by regional lymphadenopathy and marked FDG uptake on PET-CT, strongly suggested a malignant disease. However, histopathologic examination confirmed granulomatous inflammation rather than malignancy. To the authors' knowledge, this is the first veterinary report describing the CT and PET-CT findings of granulomatous hepatitis in a dog. This case describes multimodality imaging features of canine granulomatous hepatic disease, including PET-CT for monitoring metabolic activity and treatment response over time.

In this case, multiple target-like lesions were observed in both the liver and spleen, a finding that is commonly considered suggestive of malignant diseases, including sarcomas, carcinomas, and round cell tumors (16). However, similar imaging appearances, although less frequently reported, have also been described in benign or inflammatory conditions such as nodular hyperplasia, pyogranulomatous hepatitis, chronic hepatitis, and hepatic cirrhosis. In human patients with granulomatous hepatitis, hyperechoic periportal bands and hyperechoic nodules surrounded by hypoechoic rims have been reported as characteristic ultrasonographic features (17). The target-like lesions in granulomatous hepatitis is thought to reflect histopathologic organization of granulomatous inflammation (18, 19). Granulomatous lesions are composed of compact aggregates of epithelioid histiocytes, often accompanied by central necrosis or poorly perfused inflammatory cores, which may correspond to relatively hyperechoic central regions on ultrasonography. These central components are surrounded by a peripheral cuff of lymphocytes, plasma cells, and fibroblasts, forming a layered structure that appears as a hypoechoic rim. This histologic heterogeneity can produce the layered ultrasound features, with a relatively hypovascular or necrotic central region surrounded by an inflammatory, hypervascular peripheral rim.

Triple-phase CT demonstrated the contrast enhancement patterns showing the internal heterogeneity of the granulomatous lesions. In the arterial phase, conspicuous peripheral enhancement was observed along the vascular margins, reflecting hyperperfusion and active inflammatory changes within the rim of the lesion. In contrast, the central regions of the lesions appeared relatively hypoattenuating during this phase, suggesting necrosis or hypoperfusion. On equilibrium-phase images, these central regions became hyperattenuating relative to the surrounding liver parenchyma. This enhancement pattern is suggestive of fibrotic tissue and reflects delayed contrast accumulation within granulomatous lesions (20). In both dogs and humans, reported CT findings of granulomatous hepatitis are limited. Pre-contrast CT findings have included irregular hepatic margins in a single veterinary case report and a lobulated hepatic contour, hypodense bands, and parenchymal septations in human patients (21, 22). However detailed contrast enhancement patterns according to time changes have rarely been described. Only one case reported irregular hypodense lesions adjacent to the hepatic veins on contrast-enhanced CT (23). In the study, dynamic MRI was additionally performed and revealed early peripheral rim enhancement followed by delayed centripetal filling, a pattern closely resembling a target-like configuration. Although these findings were described on MRI rather than CT, this delayed centripetal enhancement pattern is compatible with the triphasic CT enhancement pattern observed in our case.

In the present case, marked regional lymphadenopathy accompanying the hepatic and splenic lesions raised a suspicion of malignancy on ultrasound and CT examinations. Therefore, PET-CT further assessed the biological behavior of the lesions using ^18^F-FDG. The hepatic lesions and lymph nodes also showed hypermetabolism. In some target lesions, the distribution of SUVmax suggested that FDG uptake was more pronounced at the periphery than at the center, with the rim demonstrating more clearly increased SUVmax values, indicating more active FDG accumulation in the peripheral portion than in the central region.

In the quantitative analysis, the hepatic lesions demonstrated a markedly elevated SUVmax of 11.2. Normal hepatic FDG uptake is typically low and relatively homogeneous under physiologic conditions. Previous studies have reported SUVmax values of approximately 1.96 for the normal canine liver and around 2.73 for normal human liver (24, 25). Therefore, markedly elevated FDG uptake detected in our case generally raises strong suspicion for a malignant process. A SUVmax value of 2.9 for the hepatic lymph node was considered slightly higher when compared with the cutoff value of 2.4 proposed in a human melanoma study for distinguishing malignant from benign lymph nodes (26). However, given the known overlap in SUVmax values between benign and malignant lymph nodes, this finding should be interpreted with caution.

SUV values tend to be higher in malignant lesions than in benign ones, and vary according to tumor type (23). On this basis, several studies in human medicine have attempted to use SUV measurements to further characterize and subclassify tumors based on their metabolic behavior. A recent human study has proposed an SUVmax cutoff value of approximately 4.9 for hepatocellular carcinoma (27). In contrast, reports of FDG uptake values for primary hepatic tumors in dogs are very limited. Accordingly, the markedly elevated SUV uptake observed in this case supported the initial suspicion of malignancy.

However, false-positive high FDG uptake in inflammatory lesions have been reported in human medicine and in a few veterinary cases with conditions such as abscesses, fungal granulomas, nodular lymphoid hyperplasia, sarcoidosis, and inflammatory pseudotumor (12–14, 28). Active inflammation can increase metabolic activity of inflammatory cells, including macrophages, neutrophils, and fibroblasts, which show high glycolytic activity and increased FDG accumulation. These falsely increased uptakes reduce the specificity of PET-CT in distinguishing malignant from benign lesions. Therefore, high SUV values should not be interpreted as definitive evidence of malignancy, particularly in the presence of active inflammatory disease. In the present case, although the SUVmax of 11.2 was markedly elevated, this degree of FDG uptake was considered high but not atypical for an active granulomatous inflammatory process, as similar or even higher SUV values have been reported in inflammatory hepatic lesions.

The marked FDG uptake observed in the present case may also have been influenced by the severe systemic inflammatory response. Previous studies have demonstrated a positive association between elevated CRP concentrations and increased FDG accumulation in inflammatory lesions, reflecting enhanced glucose metabolism of activated inflammatory cells (29, 30). At presentation, the dog's CRP concentration was markedly increased (183 mg/L), supporting the presence of active inflammation.

Importantly, serial PET-CT proved useful for longitudinal assessment of metabolic activity. In contrast to CT and ultrasonography, which primarily depict morphologic changes, PET-CT provides complementary metabolic information that may help assess disease activity and treatment response over time. On repeat PET-CT 4 months later, the hepatic lesions showed a reduction in SUVmax from 11.2 to 8.8. This decrease in FDG uptake over time likely reflected reduced metabolic activity of inflammatory cells rather than true disease regression in the setting of chronic inflammatory disease (31). This metabolic trend was subsequently accompanied by interval ultrasonographic changes of the liver, including progressive parenchymal alteration and morphologic features consistent with chronic hepatic disease. These findings suggest that serial PET-CT may be useful for monitoring metabolic changes before or alongside detectable morphologic alterations in chronic inflammatory hepatic disease (32).

This case report has several limitations. First, the diagnosis of granulomatous hepatitis lesion was based on percutaneous Tru-cut biopsy samples, which may not fully represent the heterogeneous characteristics of the entire lesion. Second, although special stains for infectious agents were negative, comprehensive microbial testing such as culture or polymerase chain reaction was not performed. Therefore, a potential infectious trigger cannot be completely excluded. Lastly, advanced hepatic fibrosis and cirrhosis were present concurrently and may have confounded imaging characteristics, metabolic uptake, and biochemical alterations, making complete separation of the granulomatous hepatitis from the underlying chronic hepatopathy challenging.

This study indicated that granulomatous hepatitis in dogs may present with multifocal hepatic lesions showing characteristic target-like appearances on ultrasonography and heterogeneous enhancement patterns on multiphasic CT. Importantly, these lesions can also demonstrate markedly increased FDG uptake on PET-CT, reflecting high metabolic activity associated with active granulomatous inflammation rather than indicating malignancy alone. Therefore, this case highlights the potential role of PET-CT beyond malignant tumor evaluation, suggesting its utility for assessing metabolic activity and monitoring therapeutic response over time in inflammatory diseases such as active granulomatous hepatitis.

## Data Availability

The raw data supporting the conclusions of this article will be made available by the authors, without undue reservation.
